# Baffled‐flow culture system enables the mass production of megakaryocytes from human embryonic stem cells by enhancing mitochondrial function

**DOI:** 10.1111/cpr.13484

**Published:** 2023-04-23

**Authors:** Xumin Wu, Bowen Zhang, Keyi Chen, Jiahui Zhao, Yunxing Li, Jisheng Li, Chuanli Liu, Lijuan He, Tao Fan, Chao Wang, Yan Li, Xuetao Pei, Yanhua Li

**Affiliations:** ^1^ School of Pharmacy Guizhou University Guiyang China; ^2^ Stem Cell and Regenerative Medicine Lab Beijing Institute of Radiation Medicine Beijing China; ^3^ South China Research Center for Stem Cell & Regenerative Medicine, SCIB Guangzhou China; ^4^ College of Chemistry and Environmental Science Hebei University Baoding China; ^5^ School of Life Science Hebei University Baoding China

## Abstract

Human embryonic stem cells (hESCs) have become an ideal cell source for the ex vivo generation of megakaryocyte (MK) and platelet products for clinical applications. However, an ongoing challenge is to establish scalable culture systems to maximize the yield of stem cell‐derived MKs that release platelets. We defined a specific dynamic 3D manufacturing system in a baffled‐flow manner that could remarkably facilitate megakaryopoiesis and increase the yield of platelet‐producing MKs from hESCs within a 12‐day induction period. Additionally, an increased number of >16N ploidy MKs, proplatelets, and platelets were generated from induced cells harvested on Day 12 using the specific dynamic culture method. The specific dynamic culture method significantly enhanced endothelium‐to‐haematopoietic transition and early haematopoiesis. More importantly, MK fate was significantly facilitated in a specific dynamic manner during early haematopoiesis. Mechanistically, this dynamic culture significantly enhanced mitochondrial function via the oxidative phosphorylation pathway and caused differentiation skewing of hESCs toward megakaryopoiesis. This study can aid in the automatic and scalable production of MKs from stem cells using baffled‐flow bioreactors and assist in the manufacturing of hESC‐derived MK and platelet products.

## Graphical abstract

Human embryonic stem cells (hESCs) have become an ideal cell source for ex vivo generation of megakaryocyte (MK) and platelet products for clinical applications. We defined a specific dynamic culture system in a baffled‐flow manner that enabled mass production of MKs from hESCs within 12 days. The specific dynamic culture method significantly enhanced the endothelium‐to‐haematopoietic transition, early haematopoiesis and MK fate specification. Mechanistically, this dynamic culture significantly enhanced mitochondrial function via the oxidative phosphorylation pathway and caused differentiation skewing of hESCs toward megakaryopoiesis. This study could aid in the automatic and scalable production of MKs from stem cells using baffled‐flow bioreactors, and could also assist in the manufacturing of hESC‐derived MK and platelet products.

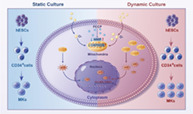

## 1 INTRODUCTION

Platelet transfusion is a globally applied approach for managing patients at risk of haemorrhage.^1^ However, the on‐demand application of platelet transfusion is often limited by the low stock availability and the short shelf life of platelets (PLTs).^2^ There exists an alternative strategy to ex vivo engineered manufacturing of induced PLTs or their precursor megakaryocytes (MKs) from stem cell differentiation or somatic cell reprogramming strategies.^3^, ^4^ Accumulated evidence indicates that stem cell‐derived MKs or PLTs can be developed as advanced therapeutic and medicinal products for patients with thrombocytopenia.^4^, ^5^, ^6^, ^7^

Human pluripotent stem cells, including human embryonic stem cells (hESCs) and induced pluripotent stem cells (hiPSCs), are ideal seed cells for the generation of therapeutic cell products because of their indefinite expansion and three‐germ‐layer differentiation capacity.^8^, ^9^ Accumulating evidence has shown that hESCs or hiPSCs can be induced to differentiate into MKs and PLTs using stromal cell co‐cultures or embryoid body induction strategies.^10^, ^11^, ^12^ Additionally, some studies have reported that gene modification by overexpressing certain transcription factors, including FLI1, MEIS1, c‐MYC, BMI1 and BCL‐XL, in pluripotent stem cells significantly enhances the output of induced MKs and PLTs.^13^, ^14^, ^15^, ^16^, ^17^ However, scalable manufacturing of MKs or PLTs from stem cells remains a challenge.

In our previous study, we developed an optimal, stepwise, three‐dimensional (3D) sphere‐like differentiation protocol for MK production by using a polystyrene CellSTACK culture chamber.^18^ However, this static 3D induction protocol can be improved by modulating the homogeneity of cell spheres during the induction process and increasing the yield of MKs from stem cells. For scalable production of cell products, it is better to establish a dynamic 3D culture system with enhanced dissolved oxygen content in the medium to improve cellular energy metabolism and expansion potential.^19^, ^20^ Several studies have indicated that disposable orbital rotary bioreactors with baffled‐flow enable large‐scale production of mammalian suspension cell cultures.^21^, ^22^ Therefore, it is valuable to evaluate whether such a dynamic culture system with baffled flasks and an orbital rotator can improve the manufacture of MKs derived from hESCs.

In this study, we established a dynamic culture system consisting of baffled flasks and an orbital rotator and demonstrated that the baffled‐flow culture system enabled the mass production of MKs from hESCs. This dynamic culture condition enhanced MK fate during the early haematopoiesis of hESCs. Mechanistically, this dynamic culture manner remarkably enhanced mitochondrial function of induced cells via the oxidative phosphorylation (OXPHOS) pathway and caused a skewing of hESCs towards megakaryopoiesis.

## 2 MATERIALS AND METHODS

### 2.1 Cell culture and differentiation

H1 and H9 hESCs (from Wicell Research Institute) were maintained on hESC‐qualified Matrigel in mTeSR1 medium, as previously described.^23^ The medium was changed daily and confluent cultures were passaged every 4–6 days using TrypLE Select. hESCs were induced to differentiate into MK lineages under chemically defined conditions.^18^ On Day 0, hESCs were dissociated into single cells and resuspended in stage I induction medium containing Advanced DMEM/F‐12 medium supplemented with 1 × GlutaMAX, 50 μg/mL l‐ascorbic acid 2‐phosphate (AA2P), 25 ng/mL BMP4, 25 ng/mL Activin A, 25 ng/mL bFGF, 2 μM CHIR99021 and 10 μM Y27632. The cells were plated at a density of 0.5–1 × 10^5^ cells/mL in baffled Erlenmeyer flasks. On Day 2, cell aggregates were collected and resuspended in stage II induction medium containing Advanced DMEM/F‐12 medium supplemented with 1 × GlutaMAX, 50 μg/mL AA2P, 50 ng/mL VEGF, 20 ng/mL bFGF and 2 μM SB431542. On Day 6, the medium was replaced with stage III induction medium containing BEL medium with 50 ng/mL SCF, 40 ng/mL TPO, 20 ng/mL IL‐3, 20 ng/mL Flt3‐L, 20 ng/mL VEGF, 20 ng/mL IGF‐1, 10 ng/mL bFGF, 20 ng/mL IL‐11 and 5 μM SB431542. The medium was changed daily during induction. The BEL medium was prepared according to the references (Table S1).^18^, ^24^ On Day 12, suspended single cells were collected by passing through a 70‐μm cell strainer for subsequent analysis. In some experiments, purified CD34^+^ cells from Day 6 of induction were isolated using magnetic‐activated cell sorting (MACS), according to the manufacturer's instructions.

For MK maturation, suspended cells from Day 12 were cultured in stage IV induction medium consisting of BEL medium supplemented with 50 ng/mL SCF, 200 ng/mL TA‐316, 5 μM diMF, 25 μM Q‐VD‐Oph, 2.5 μM harmine and 25 ng/mL CCL5 for 3 days. On Day 15, the medium was changed to stage V induction medium consisting of BEL medium supplemented with 50 ng/mL SCF, 200 ng/mL TA‐316, 10 μM KP‐457, 0.75 μM SR1, 10 μM Y27632, 10 U/mL Heparin and 1% chemically defined lipid concentrate. MKs and PLTs were collected 3 days later for various analyses. The main reagents used for hESC culturing and differentiation are listed in Table S2.

### 2.2 Flow cytometry analysis

For cell‐surface antigen analysis, cells were harvested, washed once with phosphate‐buffered saline (PBS), and further incubated with antibodies in PBS for 40 min at 4°C in the dark, following the manufacturer's instructions and suggested dilutions. For platelet analysis, the pellet in the culture supernatant was resuspended in HEPES‐buffered Tyrode's solution containing 1 μM PGE_1_ and then stained with 2 μg/mL Calcein AM for 40 min at 37°C. After washing twice with buffer, the pellet was stained with antibodies against CD41a and CD42b for 15 min at 37°C in the dark. Stained cells or PLTs were analysed using FACS Aria II (BD) or Guava easyCyte (Luminex), and the data were analysed using FlowJo software. The sources of antibodies and fluorescent probes are listed in Table S2.

### 2.3 Quantitative PCR analysis

Differentiated cells were collected and total RNA was isolated using a RNeasy mini kit and quantified using a NanoDrop spectrophotometer (Thermo). Equal amounts of RNA were reverse‐transcribed using ReverTra Ace™ qPCR RT Master Mix, according to the manufacturer's instructions. Quantitative RT‐PCR (RT‐qPCR) was performed using SYBR™ Green PCR Master Mix on a Real‐Time PCR detection system (Bio‐Rad). The sample input was normalized to the cycle threshold (Ct) value for *GAPDH*. The primers used are listed in Table S3.

### 2.4 Immunofluorescence assay

Cells were fixed with 4% paraformaldehyde for 20 min at 25°C, permeabilized in PBS with 0.2% Triton X‐100 for 10 min, and blocked in PBS containing 10% donkey serum for 1 h. The cells were then incubated with diluted primary antibodies in PBS with 10% donkey serum at 4°C overnight, followed by incubation with the corresponding secondary antibodies in the dark at room temperature for 30 min. The cells were washed three times with PBS between each step. Cell nuclei were counterstained with 4′,6‐diamidino‐2‐phenylindole (DAPI) before visualization and image acquisition using a CSIM 100 confocal scanning imaging system (Sunny). Specific information regarding the antibodies used is listed in Table S2.

### 2.5 RNA‐sequencing and analysis

Samples from three independent biological experiments were used and the RNA‐sequencing was performed by the BGI Company (BGI, Shenzhen, China). All analyses, including gene ontology (GO) enrichment, Kyoto Encyclopedia of Genes and Genomes (KEGG), gene set enrichment analysis (GSEA) and heatmap analysis, were performed using the online bioinformatics platform Dr. Tom provided by BGI. The data are available at Gene Expression Omnibus (GEO) (Accession number: GSE228163)

### 2.6 Colony‐forming unit assay

For haematopoietic colony‐forming unit (CFU) assay, cells were washed, counted, resuspended in Iscove's Modified Dulbecco's Medium (IMDM), added to MethoCult™ SF H4636, and plated at a density of 5 × 10^3^ cells/well in ultra‐low attachment 24‐well plates. After 12 days of culture, colonies were counted and scored by morphology as erythroid (BFU‐E, burst‐forming unit‐erythroid), myeloid (CFU‐GM, CFU‐granulocyte, monocyte) or mixed lineage (CFU‐GEMM, CFU‐granulocyte, erythrocyte, monocyte and megakaryocyte).

For myeloid and MK colony analysis, cells were resuspended in 300 μL of IMDM medium and added to 3.3 mL of MethoCult^TM^ H4535 supplemented with 50 ng/mL SCF and 100 ng/mL TPO and plated at a density of 1 × 10^4^ cells/well.^17^ After 12 days of culture, single colonies were cytospun onto slides and subjected to Giemsa staining and CD41 antibody staining. Images were captured using a microscope (Nikon).

### 2.7 CFU‐MK assay

To detect and quantify CFU‐MK, 3 × 10^3^ cells/well of differentiated cells were plated in a collagen‐based semi‐solid medium using the MegaCult‐C complete kit. Cells were cultured at 37°C in a 5% CO_2_ incubator. On Day 14, MK colonies were fixed and stained with anti‐CD41 antibody, according to the manufacturer's instructions. CD41^+^ colonies were counted and categorized.

### 2.8 O_2_ measurement

An oxygen microsensor (OXY‐1 ST Trace) was used to periodically record the level of dissolved oxygen in the medium. Briefly, 1 mL of culture medium was transferred into an Eppendorf tube. A microsensor was inserted below the liquid level, and the measured value was recorded a few minutes after it stabilized.

### 2.9 Factor V uptake assay

For Factor V (FV) uptake assays, differentiated cells were cultured in BEL medium supplemented with 30 nM FV‐Atto‐488 for 18 h at 37°C.^25^, ^26^ FV‐treated cells were collected, washed, and then stained with CD41a and CD42b antibodies for 30 min at 4°C. Images were captured using a microscope (Nikon). Stained cells were analysed using flow cytometry.

### 2.10 Ploidy analysis

For polyploidy analysis, the cultured cells were collected and resuspended in PBS containing 5 μg/mL Hoechst 33342 and then labelled with CD41a and CD42b antibodies for 30 min before flow cytometry.

### 2.11 Statistical analysis

All data are presented as means ± SEM. Statistical analyses were performed using GraphPad Prism 8 software. Statistical significance of differences was determined using unpaired two‐tailed Student's *t*‐test or Ordinary one‐way ANOVA. Significance was assumed at *p < 0.05, **p < 0.01.

## 3 RESULTS

### 3.1 Baffled‐flow culture system enables mass production of MKs from hESCs

**FIGURE 1:**
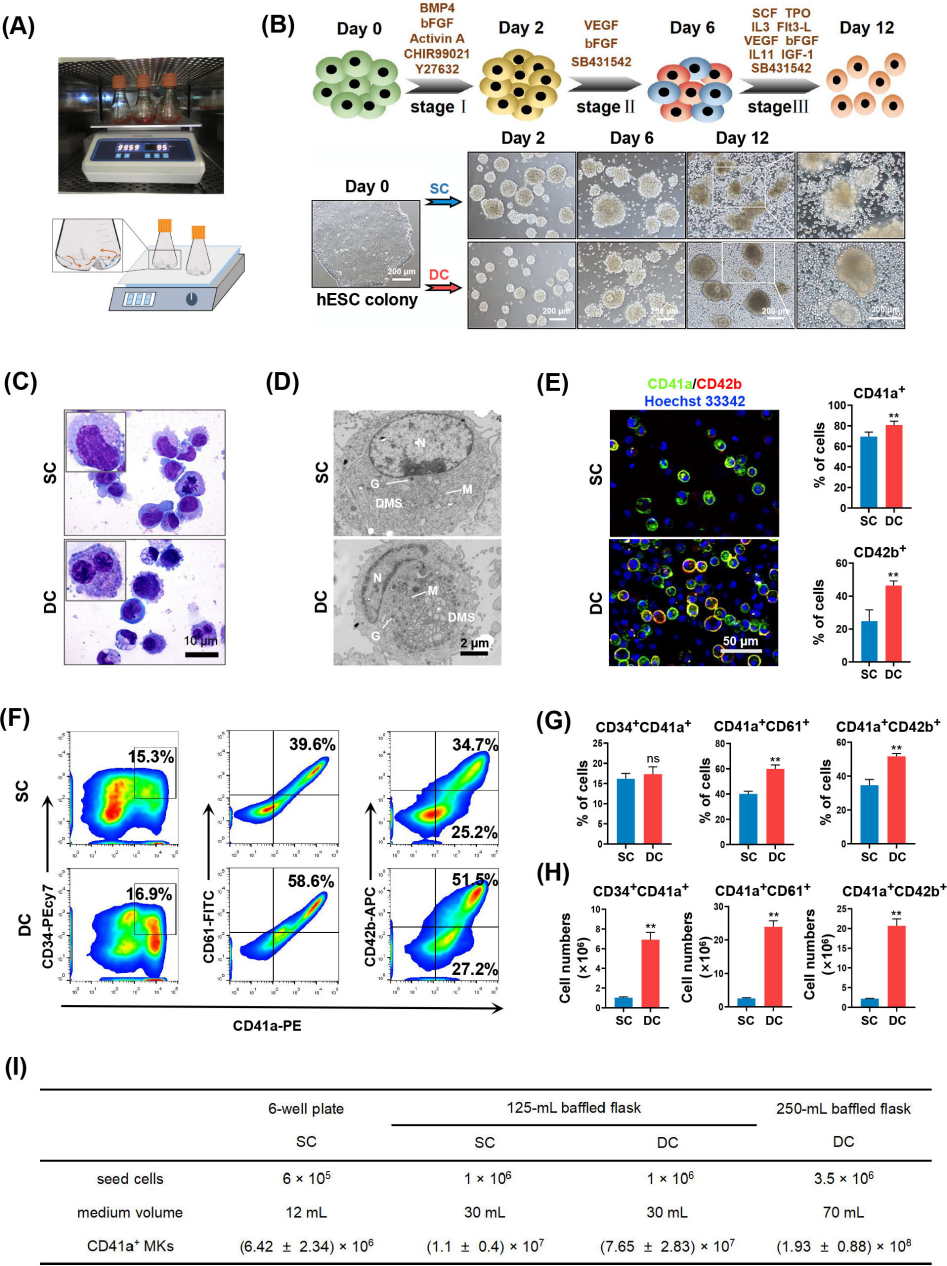
Large‐scale generation of hESC‐derived MKs through baffled‐flow culture system. (A) Illustration of the baffled‐flow culture system. (B) Schematic representation of differentiation of hESCs towards MKs *in vitro*. Phase contrast images of cells during differentiation from hESCs to MKs in SC and DC groups on Day 2, Day 6 and Day 12. Scale bars represent 200 μm. (C) Giemsa staining of suspended single cells from SC or DC groups of MK differentiation on Day 12. Scale bars represent 10 μm. (D) Transmission electron microscopy images of hESC‐derived MKs from SC and DC groups on Day 12. N, nucleus; M, mitochondria; G, granules; DMS, demarcation membrane system. Scale bars represent 2 μm. (E) Staining graph of live cells with CD41a (green), CD42b (red) and Hoechst 33342 and the percentage of CD41a^+^ and CD42b^+^ cells in the two groups on Day 12. Five random microscopic fields were counted for each group. Scale bars represent 50 μm. (F) Representative flow cytometry results of MK markers (CD34, CD41a, CD61 and CD42b) in suspension cells from SC and DC groups on Day 12. (G, H) Bar graph showing the percentage and number of CD34^+^CD41a^+^, CD41a^+^CD61^+^ and CD41a^+^CD42b^+^ cells from SC and DC groups on Day 12. *n* = 3. (I) The yields of H9 hESC‐derived CD41a^+^ MK was analysed using six‐well plates, 125‐mL flasks and 250‐mL flasks. Data are shown for three independent experiments. Experiments were performed on H1 hESCs unless otherwise indicated.

A baffled‐flow culture system with the advantage of increased medium aeration has been used for the scalable propagation of mammalian suspension cells.^22^ To improve the scalable production of MKs from hESCs in a bioreactor‐like system, we established a dynamic 3D culture protocol using baffled flasks on an orbital rotator (Figure 1A). Briefly, hESC colonies were digested into single cells and transferred into flasks for haematopoietic and MK differentiation in a baffled‐flow culture pattern for 12 days (Figure 1B). We first compared the yield of MKs generated from hESCs in flasks with or without a baffle structure under dynamic culture conditions. Baffled‐flow conditions resulted in higher yields of CD41a^+^ and CD41a^+^ CD42b^+^ cells derived from hESCs than unbaffled‐flow conditions on Day 12 (Figure S1A,B). We then tested the influence of different rotary speeds (60, 95 and 130 rpm) driven by a 10‐mm orbital rotator in the baffled‐flow system on the production of MKs from hESCs. Static culture (SC) conditions, as controls, were manipulated in baffled flasks without agitation. We found that the yield of MKs derived from hESCs was highest at 95 rpm (Figure S1C). Thus, we adopted a dynamic culture system with a baffled‐flow design at a speed of 95 rpm driven by a 10‐mm orbital rotator and defined it as the specific dynamic culture (DC) condition for MK production.

The specific DC condition led to the formation of relatively smaller and more uniform cell spheres derived from single hESC than the SC condition (Figure 1B). Wright‐Giemsa staining demonstrated the presence of large or multinuclear cells with granular cytoplasm on day 12 after SC or DC morphologically assembled with MKs (Figure 1C). Transmission electron microscopy of single suspension cells collected on Day 12 demonstrated typical MK organelles, including a demarcation membrane system (DMS) and granules (G) (Figure 1D). More single‐cell suspensions were generated around these cell spheres under DC conditions on Days 6 and 12 (Figure 1B). Consistently, a substantially increased number of CD41a‐ and CD42b‐positive cells were observed in suspended single cells using the living cell staining technique (Figure 1E). Flow cytometry results showed that the proportions of CD34^+^CD41a^+^, CD41a^+^CD61^+^ and CD41a^+^CD42b^+^ cells were 17.3 ± 1.83%, 59.9 ± 3.16% and 51.7 ± 1.66%, respectively, under DC conditions (Figure 1F,G). The DC system substantially increased the yield of CD34^+^CD41a^+^, CD41a^+^CD61^+^ and CD41a^+^CD42b^+^ cells compared to SC conditions (Figure 1H). We also manipulated the MK induction process in a 250‐mL baffled flask. Starting from 3.5 × 10^6^ H9 hESCs in a single 250‐mL flask, we obtained a yield of (1.93 ± 0.88) × 10^8^ CD41a^+^ MKs after 12 days of induction. Compare with the MK yield in a 6‐well plate, the DC method in a 250‐mL baffled flask led to 30.1‐fold increase in MK yield (Figure 1I). These data indicate that the 3D DC system with baffled‐flow enabled the mass production of MKs from hESCs.

### 3.2 Baffled‐flow culture system enhances megakaryopoiesis of hESCs

**FIGURE 2:**
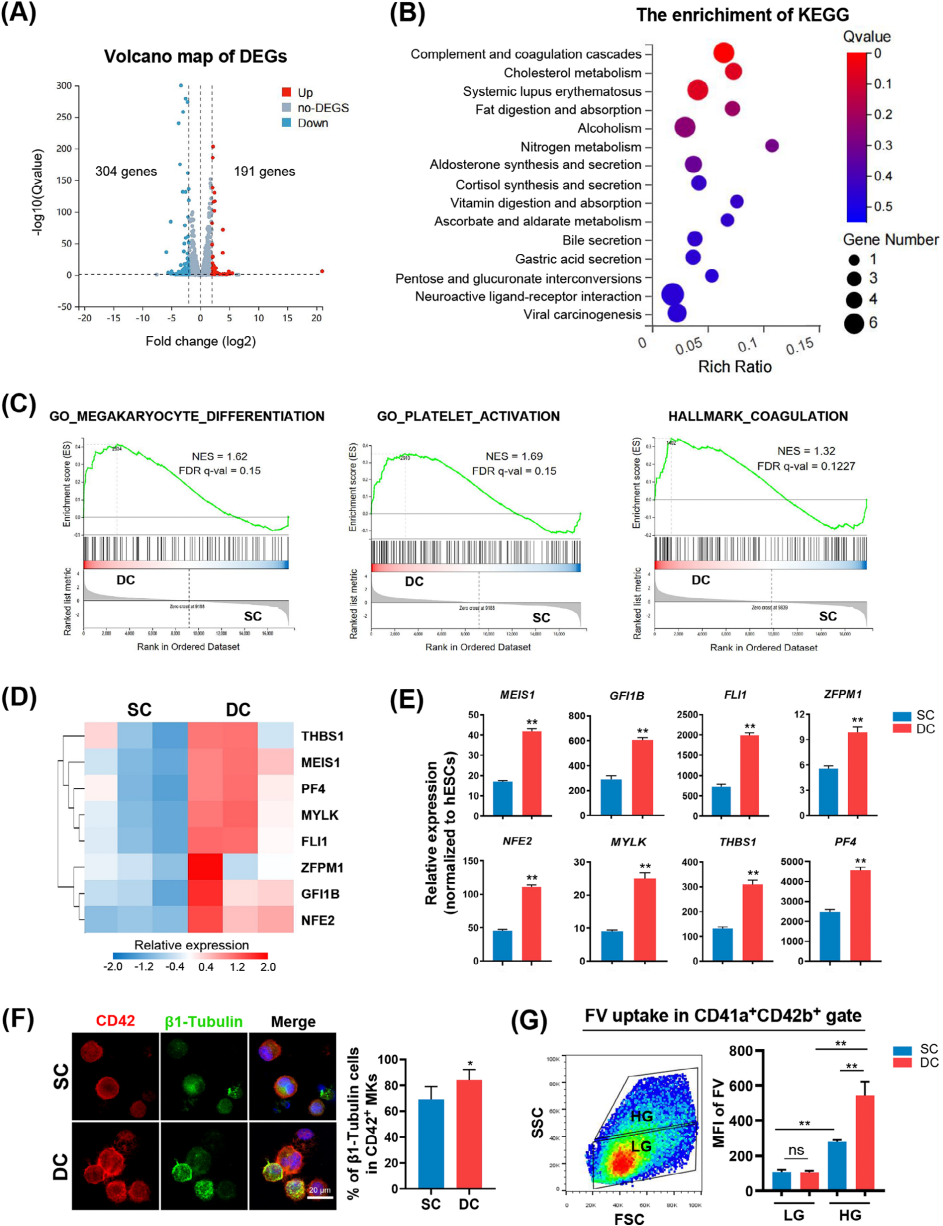
Enhancing megakaryopoiesis in hESCs using a baffled‐flow culture system. (A) Volcano map displaying DEGs of CD41^+^ cells between SC and DC groups on Day 12. (B) KEGG analysis showing the top terms enriched by the upregulated genes in DC groups compared to SC groups on Day 12. The size of each dot is based on the number of genes enriched in the pathway, and the colour of the dots represents the significance of pathway enrichment. (C) GSEA comparing cells between SC and DC groups for MK differentiation, platelet activation and coagulation gene set. (D) Heatmap illustrating relative expression of MK‐related genes on Day 12. (E) RT‐qPCR analysis of MK‐related genes in SC and DC groups on Day 12. Relative expression levels were normalized to hESC levels. *n* = 3. (F) Representative images of CD42 (red) and β1‐Tubulin (green) staining in hESC‐derived MKs. The bar graph indicates the percentage of β1‐Tubulin^+^ cells in CD42b^+^ cells. Five random microscopic fields were counted for each group. Scale bars represent 20 μm. (G) Representative size (FSC) and granularity (SSC) profiles of CD41a^+^CD42b^+^ cells. Flow cytometric gating of low granular (LG) and high granular (HG) MKs is displayed. The bar indicates the mean fluorescence intensity (MFI) of FV uptake of LG and HG in CD41a^+^CD42b^+^ cells. *n* = 3. Experiments were performed on H1 hESCs.

To investigate whether megakaryopoiesis was enhanced in DC conditions, RNA sequencing (RNA‐seq) analysis was performed using the generated suspended cells under SC or DC conditions on Day 12 of induction. A total of 495 differentially expressed genes (DEGs) were identified in the DC group compared with those in the SC group (Figure 2A). Among them, 191 DEGs, including *PF4* and *NFE2*, were significantly upregulated and 304 DEGs were downregulated in the DC group (Figure 2A). Genes in the DC group were strongly enriched for KEGG terms related to complement and coagulation cascades (Figure 2B). Additionally, GSEA showed that the induced cells generated in the DC group were enriched in genes related to MK differentiation, platelet activation, and coagulation (Figure 2C). Consistently, MK‐related genes, such as *FLI1*, *NFE2*, *THBS1* and *PF4* were significantly upregulated in the DC group (Figure 2D). We then performed RT‐qPCR to determine the MK gene expression levels under different culture conditions. The results showed that the DC system increased the expression of MK‐related genes, including *MEIS1*, *GFI1B*, *THBS1* and *PF4* (Figure 2E). Immunofluorescence staining showed that the DC system caused a significant increase in the percentage of β1‐Tubulin^+^ cells in the CD42^+^ MK population (Figure 2F). We then performed FV uptake experiments to evaluate the presence of platelet‐producing MKs derived from hESCs under different culture conditions.^25^, ^27^ The induced CD42b^+^ MKs were gated as low granular (LG) and high granular (HG) MKs according to their size and granularity.^25^ The induced HG CD42b^+^ MKs endocytosed FV more efficiently than the induced LG MKs, similar to a previous report (Figure 2G).^25^ HG CD42b^+^ MKs under DC conditions demonstrated enhanced FV uptake compared to those cells under SC conditions (Figure 2G). Taken together, these data indicate that the DC system with baffled flasks on an orbital rotator enhances megakaryopoiesis and increases the generation of platelet‐producing MKs.

### 3.3 Induced MKs generated from baffled‐flow culture system produced proplatelets and PLTs

**FIGURE 3:**
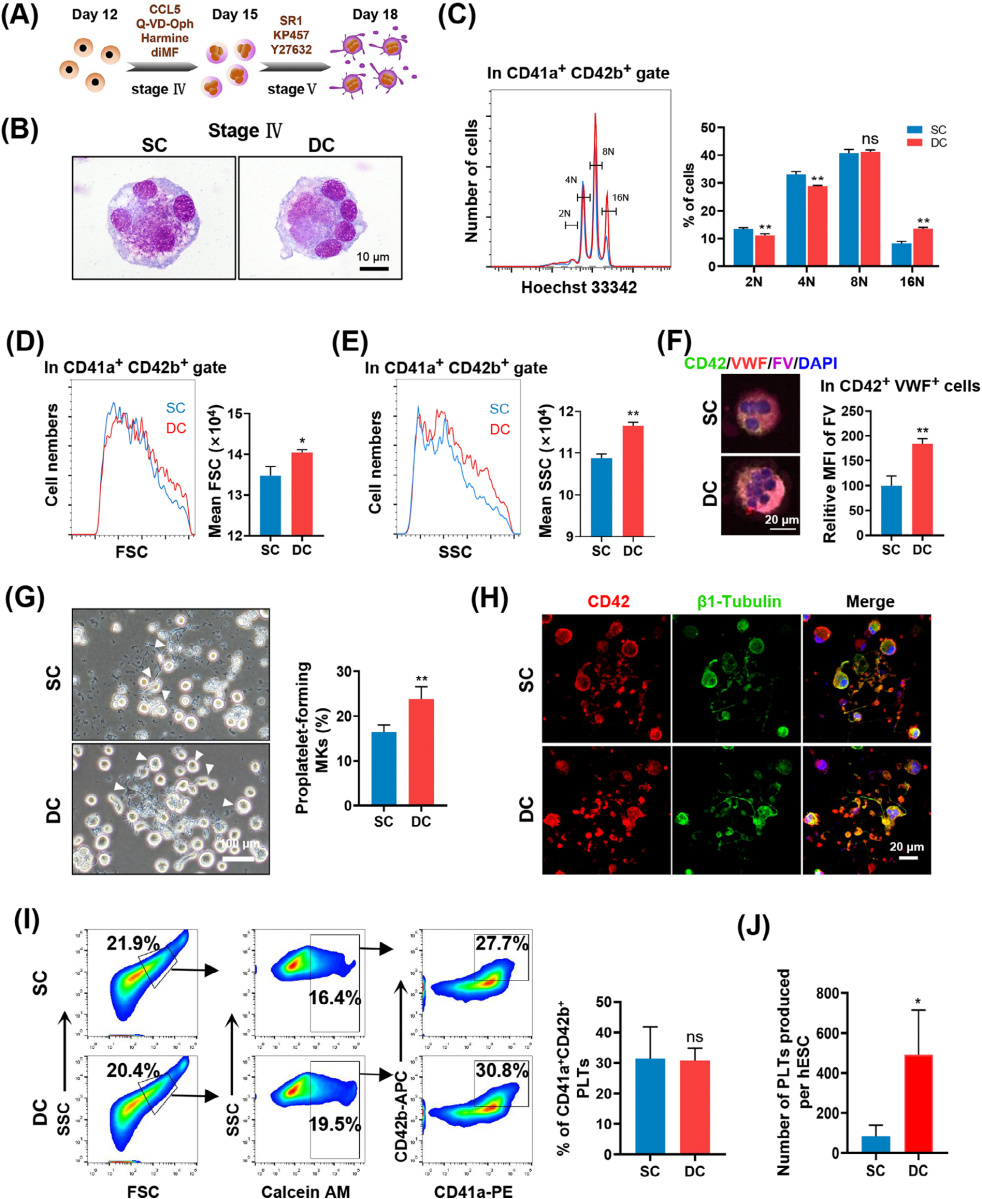
Proplatelets and platelets were produced by MKs generated from the baffled‐flow culture system. (A) Schematic representation of induction of MK maturation and platelet production from hESC‐derived MKs in two stages. (B) Giemsa staining of mature MKs from SC and DC groups on Day 15. Scale bars represent 10 μm. (C) Polyploidy analysis of MKs as assessed using Hoechst 33342 staining and flow cytometry. Representative panels and the percentage of cells with different levels of ploidy are shown. *n* = 3. (D) Size (FSC) of CD41a^+^CD42b^+^ cells. The bar graph indicates the mean FSC in CD41a^+^CD42b^+^ cells. *n* = 3. (E) Complexity/granularity (SSC) of CD41a^+^CD42b^+^ cells. The bar graph indicates the mean SSC in CD41a^+^ CD42b^+^ cells. *n* = 3. (F) Representative immunostaining images of CD42 (green), VWF (red) and factor V (purple) staining in hESC‐derived MKs on Day 15. The bar graph presents FV uptake by CD42b^+^VWF^+^ cells as measured by MFI and expressed as percentage of SC. Six random fields were counted in each group. Scale bars represent 20 μm. (G) Phase contrast images of proplatelets in SC and DC groups on Day 18. The bar graph indicates the percentage of proplatelet‐forming MKs. Five random fields were counted for each group. Scale bars represent 100 μm. (H) Representative confocal micrographs of proplatelets during PLT release on Day 18 in SC and DC groups. Scale bars represent 20 μm. (I) Flow cytometry analysis of FSC and SSC, Calcein AM and CD41a and CD42b expression on hESC‐derived PLTs. The bar graph shows the percentage of CD41a^+^CD42b^+^ cells in the Calcein AM‐positive gate on hESC‐derived PLTs. *n* = 3. (J) Bar graph indicating the number of PLTs generated by each hESC on Day 18. *n* = 3. Experiments were performed on H9 hESCs.

To observe the platelet‐producing ability of the induced MKs, 2 × 10^5^ single‐cell suspensions generated on Day 12 under DC or SC conditions were transferred to another two‐stage induction system (Stages IV and V) to accelerate MK maturation and release PLTs (Figure 3A). We found that the induced cells from the two groups could further differentiate into large polyploid MKs (Figure 3B). We analysed the nuclear content of the induced MKs at stage IV using flow cytometry and found that the DC group exhibited significantly increased 16N polyploidization (Figure 3C). Differentiated cells from the DC group exhibited increased size and complexity/granularity compared with cells from the SC group, as reflected by flow cytometry analysis of forward scatter (FSC) and side scatter (SSC) in CD41a^+^CD42b^+^ cells (Figure 3D,E). The induced CD42b^+^VWF^+^ cells from the DC group obtained at the end of stage IV showed a greater FV uptake ability than cells from the SC group (Figure 3F). These induced cells further developed into proplatelets with typical morphological features, such as multiple protrusions, thread‐like cytoplasmic extensions with bead‐like nubs, and high expression of CD42 and β1‐Tubulin (Figure 3G,H). Additionally, the induced cells from the DC group showed an increased number of proplatelets compared with those from the SC group (Figure 3G). PLTs released from these differentiated cells after two‐stage induction were detected and counted using flow cytometry. The percentage of viable PLTs expressing CD41a and CD42b was similar between the two groups on Day 18 (Figure 3I). Platelet yields derived from the initial hESCs were calculated according to the number of induced MKs obtained on Day 12 and the induced PLTs generated on Day 18. A remarkably increased yield of PLTs was achieved from induced cells in the DC group (DC: 491 ± 224 PLTs/hESC; SC: 84 ± 54 PLTs/hESC; Figure 3J). These data indicate that the dynamic baffled‐flow culture conditions enable the mass generation of MKs and PLTs.

### 3.4 DC conditions promote endothelium‐to‐haematopoietic transition and enhance early haematopoiesis of hESCs

**FIGURE 4:**
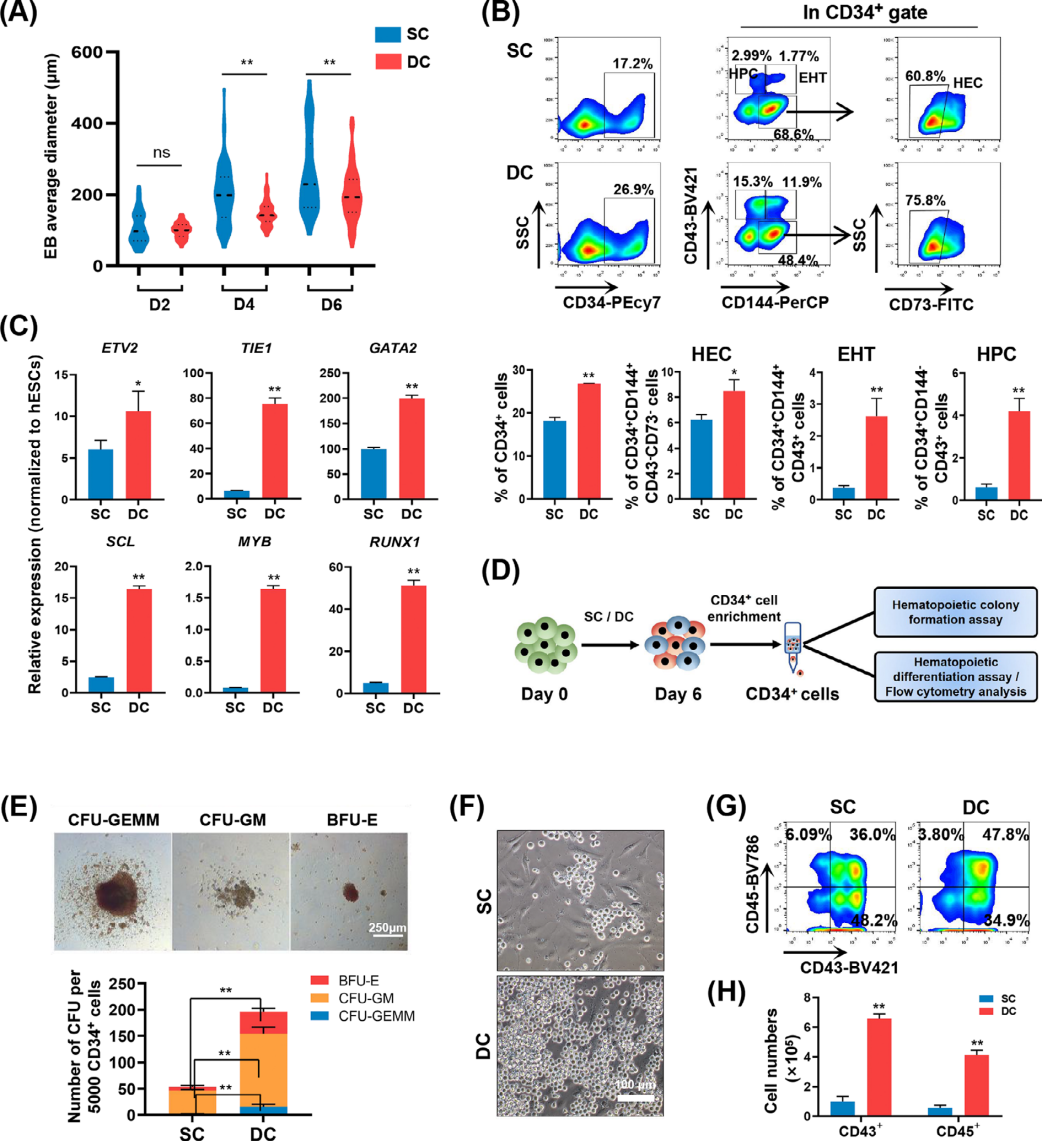
DC promotes endothelium‐to‐haematopoietic transition and enhances early haematopoiesis of hESCs. (A) Average spheroid diameter in the SC and DC groups on Days 2, 4 and 6. A total of 100 spheroids were measured for each group. (B) Representative flow cytometry results of surface markers CD34, CD43, CD144 and CD73 in the SC and DC groups on Day 6. The bar graph shows the percentage of CD34^+^ cells, CD34^+^CD43^−^CD144^+^CD73^−^ cells, CD34^+^CD43^+^CD144^+^ cells and CD34^+^CD43^+^CD144^−^ cells derived from the SC and DC groups on Day 6. *n* = 3. (C) RT‐qPCR analysis of genes associated with the endothelium and haematopoiesis. Relative expression levels were normalized to hESC levels. *n* = 3. (D) Schematic representation of the haematopoietic colony formation assay and haematopoietic differentiation assay with CD34^+^ cells. (E) Representative colony morphologies generated from hESC‐derived CD34^+^ cells. The bar graph shows the total number of colonies in the SC and DC groups. Scale bars represent 250 μm. *n* = 3. (F) Phase contrast images of haematopoietic cells after haematopoietic differentiation assays for 5 days. Scale bars represent 100 μm. (G) Representative flow cytometry results of the surface markers CD45 and CD43 after haematopoietic induction for 5 days. (H) Bar graph showing the total number of CD43^+^ and CD45^+^ cells in the SC and DC groups. *n* = 3. Experiments were performed on H9 hESCs.

To dissect how dynamic baffled‐flow culture promotes megakaryopoiesis, we examined the early differentiation process at stages I and II. No obvious differences in the expression of the mesodermal progenitor marker, apelin receptor (APLNR), were observed on Day 2 of stage I (Figure S2). Cell aggregates derived from hESCs were smaller and more uniform in the DC group than those in the SC group on Days 4 and 6 (Figure 4A). We then tracked the development of the hemogenic endothelium (HE) and early haematopoiesis. There was a significant increase in the percentages of CD34^+^ and CD34^+^CD43^−^CD144^+^CD73^−^ cells in the DC group compared to those in the SC group on Day 6 (Figure 4B), indicating that HE development was enhanced under the DC condition. Consistently, the percentage of CD34^+^CD144^+^CD43^+^ cells, representing the cell population in the endothelium‐to‐haematopoietic transition (EHT) process, was remarkably elevated in the DC group on Day 6 (Figure 4B). Moreover, the DC group showed a significantly increased proportion of CD34^+^CD144^−^CD43^+^ haematopoietic progenitor cells (HPCs) compared with the SC group (Figure 4B). The expression levels of key HE and early haematopoiesis development genes, such as *TIE1*, *GATA2*, *SCL*, *MYB* and *RUNX1*, were significantly upregulated in the DC group compared to those in the SC group (Figure 4C). We then performed CFU assays to functionally evaluate the haematopoietic potential of differentiated CD34^+^ cells enriched on Day 6 in the two groups (Figure 4D). CD34^+^ cells from the DC group showed a significant increase in CFU numbers, including CFU‐GEMM, CFU‐GM and BFU‐E, compared with cells from the SC group (Figure 4E). We also transferred enriched CD34^+^ cells into a haematopoietic differentiation medium to further assess their ability to generate haematopoietic progenitor cells (Figure 4D). After 5 days of induction, we found that more oval cells sprouted from adherent cells in the DC group (Figure 4F). Flow cytometry results revealed high proportions of CD43^+^ and CD45^+^ cells among the suspended cells from the two groups (Figure 4G). Additionally, differentiated cells from the DC group demonstrated a higher number of CD43^+^ and CD45^+^ cells than those from the SC group (Figure 4H). Collectively, these data indicate that the DC method significantly enhances the cell fate commitment of hESCs towards early haematopoiesis in stage II.

### 3.5 DC manner promotes MK‐fated haematopoiesis

**FIGURE 5:**
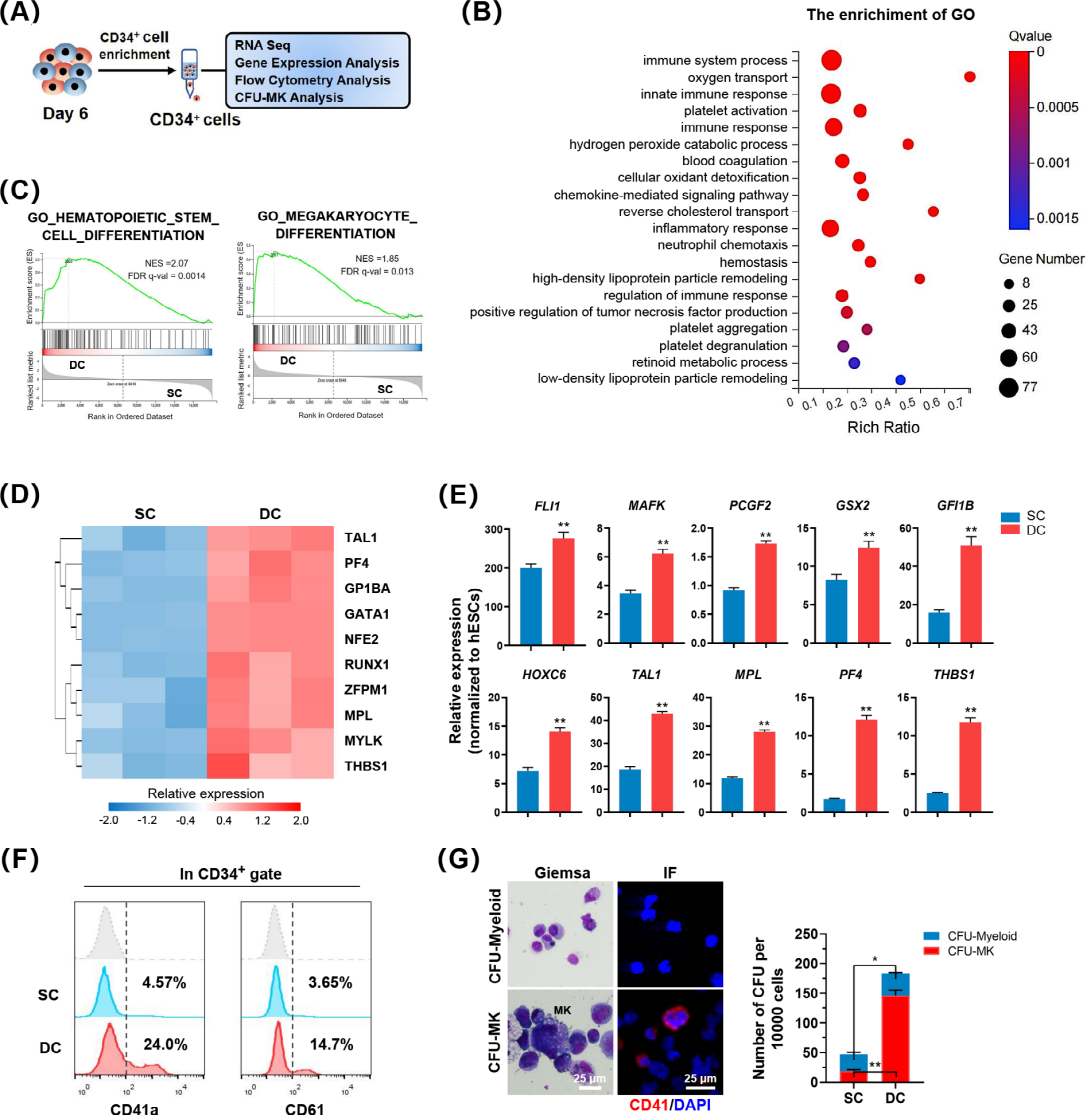
DC promotes MK‐fated haematopoiesis. (A) Schematic representation of the experiment. (B) GO enrichment analysis revealed the top terms enriched by the upregulated genes in DC groups compared to SC groups on Day 6. The size of each dot is based on the number of genes enriched in the pathway, and the colour of the dots represents the significance of pathway enrichment. (C) GSEA comparing hESC‐derived CD34^+^ cells from SC and DC groups for haematopoietic stem cell differentiation and megakaryocyte differentiation gene sets. (D) Heatmap illustrating the relative expression of the RNA‐seq data of MK‐related genes on Day 6. (E) RT‐qPCR analysis of genes associated with MKs on Day 6. Relative expression levels were normalized to hESC levels. *n* = 3. (F) Flow cytometry results of CD41a and CD61 on CD34^+^ cells in the SC and DC groups on Day 6. *n* = 3. (G) Representative morphologies of Giemsa staining (left) and CD41 immunofluorescence (IF, right) of CD34^+^ cells seeded after 12 days. The bar graph shows the number of CFU‐MK and CFU‐myeloid colonies derived from the SC and DC groups. Scale bars represent 25 μm. *n* = 3. Experiments were performed on H9 hESCs.

To investigate whether dynamic baffled‐flow culture initiates MK fate during early haematopoiesis, we performed RNA‐seq, gene expression, flow cytometry, and CFU analysis using enriched CD34^+^ cells from the two groups on Day 6 after the two‐stage induction (Figure 5A). DC conditions led to the upregulation of 1175 DEGs compared with the controls (Figure S3A). KEGG analysis showed that the genes upregulated in the DC group were enriched in pathways such as platelet activation, haematopoietic cell lineage, complement and coagulation cascades (Figure S3B). Additionally, GO analysis revealed that biological processes, including platelet activation, blood coagulation and platelet aggregation, were significantly enriched in the DC group (Figure 5B). Moreover, GSEA showed enrichment of pathways related to haematopoietic stem cell differentiation, MK differentiation, platelet degranulation and coagulation in the DC group (Figure 5C and S3C). MK‐related genes, such as *RUNX1*, *MPL*, *NFE2* and *THBS1* were significantly upregulated in the DC group (Figure 5D). RT‐qPCR results confirmed that the gene expression levels of key MK‐related transcription factors and functional genes, including *FLI1*, *PCGF2*, *GFI1B* and *THBS1*, were upregulated in the DC group compared with the SC group (Figure 5E). Moreover, the DC group presented higher percentages of CD41a^+^ and CD61^+^ cells in the CD34^+^ cell population on Day 6 (Figure 5F), suggesting enhanced MK fate during early haematopoiesis of hESCs in vitro in a DC manner. To further assess whether CD34^+^ cells from the DC group on Day 6 had strong MK development potential, CFU assays were performed with enriched CD34^+^ cells cultured in MethoCult medium supplemented with 50 ng/mL SCF and 100 ng/mL TPO.^17^ After 12 days, we scored and counted the number of CFU‐myeloid and CFU‐MK cells that contained ≥3 MKs according to the results of Wright–Giemsa and CD41 immunofluorescence staining (Figure 5G).^28^ Our results showed that cells from the DC group produced a higher proportion of CFU‐MK in the total CFUs than those from the SC group (DC vs. SC: 80.1 ± 0.19% vs. 39.6 ± 0.7%; Figure 5G), indicating that the early generated CD34^+^ cells on Day 6 in the DC group were skewed toward megakaryopoiesis. In addition, we observed an increased number of CFU‐MK and total CFUs generated by CD34^+^ cells in the DC group compared with those in the SC group (Figure 5G). Consistently, when the enriched CD34^+^ cells from the two groups were cultured in a haematopoietic differentiation medium for 5 days, higher percentages of CD34^+^CD41a^+^ and CD41a^+^CD42b^+^ cells were produced from the induced cells of the DC group compared with those in the SC group (Figure S3D). These data suggest that dynamic baffled‐flow culture significantly enhances MK fate commitment during the early stages of haematopoietic differentiation.

### 3.6 DC promotes megakaryopoiesis via enhancing mitochondrial function

**FIGURE 6:**
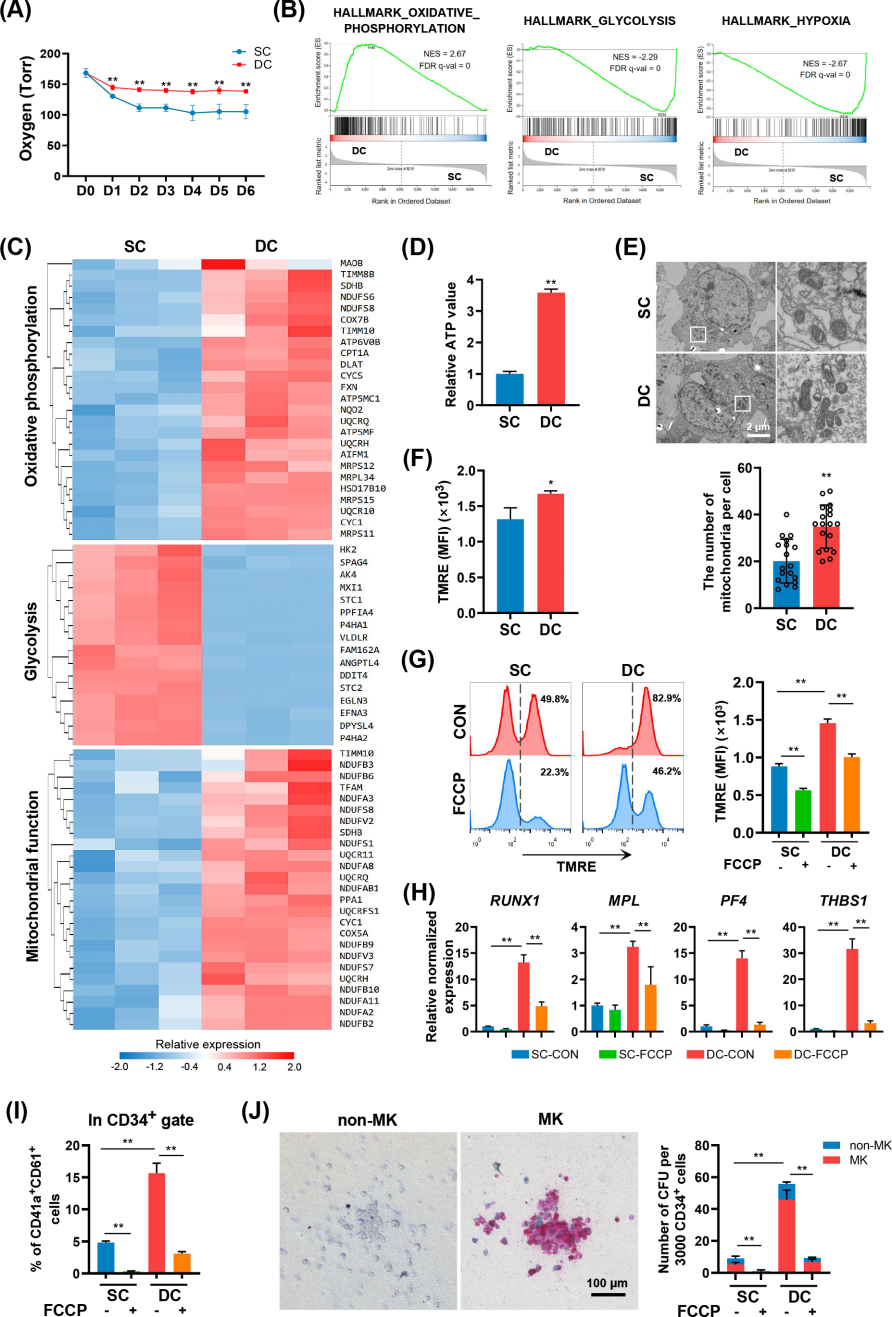
DC promotes megakaryopoiesis via improving mitochondrial activity. (A) Dissolved oxygen levels in culture medium in SC and DC groups for an incubation period of 6 days. *n* = 5. (B) GSEA comparing hESC‐derived CD34^+^ cells from SC and DC groups for oxidative phosphorylation, glycolysis and hypoxia gene set. (C) Heatmap illustrates relative expression of genes associated with oxidative phosphorylation, glycolysis, and mitochondrial function on Day 6. (D) Relative ATP value of hESC‐derived CD34^+^ cells in SC and DC groups. *n* = 3. (E) Transmission electron microscopy images of hESC‐derived CD34^+^ cells in SC and DC groups. The bar graph shows the number of mitochondria per cell. Twenty cells were counted in each group. Scale bars represent 2 μm. (F) MFI of TMRE staining for hESC‐derived CD34^+^ cells in SC and DC groups. *n* = 3. (G) Representative flow cytometry plots of TMRE staining of hESC‐derived CD34^+^ cells in SC and DC groups. The bar graph shows the MFI of TMRE staining on hESC‐derived CD34^+^ cells treated with or without FCCP (2 μM) in SC and DC groups. *n* = 3. (H) RT‐qPCR analysis of haematopoiesis‐ and MK‐related genes after 4‐day culture with or without FCCP (2 μM) in SC and DC groups on Day 6. Relative expression levels are normalized to *GAPDH* levels. *n* = 3. (I) Bar graph showing the percentage of CD41a^+^CD61^+^ in CD34^+^ cells after 4‐day culture with or without FCCP (2 μM) in SC and DC groups. *n* = 3. (J) Representative morphologies of MK and non‐MK colonies in MK colony assays after FCCP‐treated in SC and DC groups. The bar graph indicates the number of MK and non‐MK colonies in the SC and DC groups. *n* = 3. Scale bars represent 100 μm. Experiments were performed on H9 hESCs.

**FIGURE 7:**
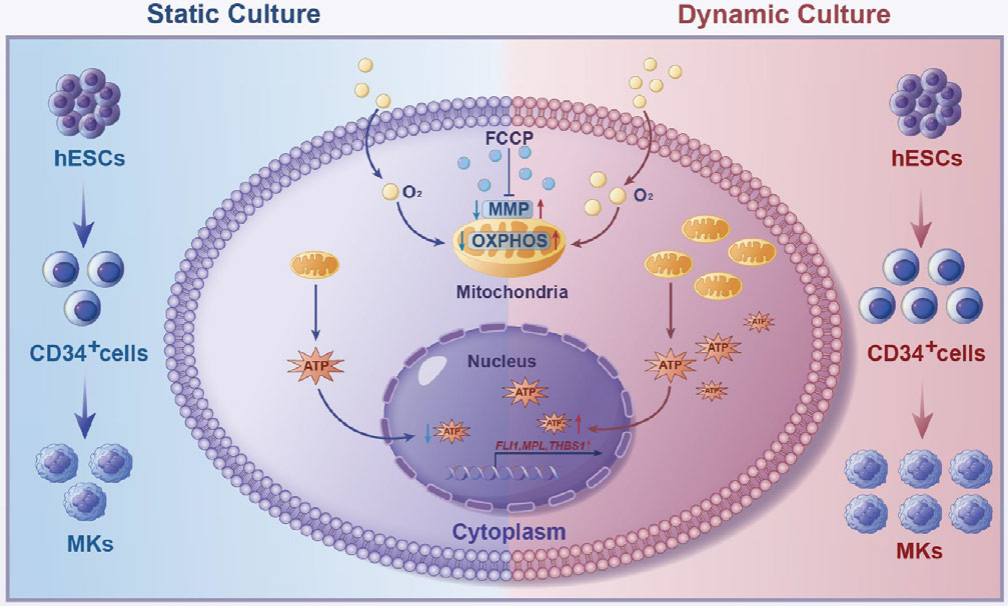
A schematic working model for the possible mechanism for DC‐enhanced megakaryopoiesis from hESCs.

To further explore the mechanism of DC in enhancing the fate of MKs, we first assessed the levels of dissolved oxygen in the medium of the two groups. Significantly elevated oxygen levels in the culture medium of the DC group were observed from Days 2 to 6 (Figure 6A). Interestingly, GSEA data revealed OXPHOS as a prominent enriched pathway in the DC group compared with that in the SC group (Figure 6B). The DC group showed significantly upregulated expression of OXPHOS‐related genes, including *COX7B*, *UQCRQ* and *FXN* (Figure 6C). In contrast, the pathways related to glycolysis and hypoxia were significantly enriched in the SC group (Figure 6B). Glycolysis‐related genes such as *HK2* and *AK4* were downregulated in the DC group (Figure 6C). In addition, the DC group showed upregulated gene expression profiles related to mitochondrial function (Figure 6C). Thus, we further analysed the levels of ATP generated in the induced cells harvested on Day 6. The DC group showed increased ATP levels in the induced cells compared with the SC group (Figure 6D), reflecting enhanced mitochondrial function in the DC group. Moreover, transmission electron microscopy of the induced cells demonstrated that the number of mitochondria was significantly higher in the DC group than in the SC group (Figure 6E). We also assessed the levels of mitochondrial membrane potential (MMP) in the induced cells using a fluorescent probe labelling method. The DC group exhibited a significantly higher MMP than the SC group (Figure 6F), indicating that a higher mitochondrial activity was generated in the DC group. Furthermore, the addition of carbonyl cyanide 4‐(trifluoromethoxy) phenylhydrazone (FCCP), which uncouples mitochondrial OXPHOS,^29^ reduced MMP in the DC and SC groups (Figure 6G). We then investigated whether MK fate‐related gene expression in the induced cells was affected by FCCP. FCCP addition significantly downregulated the expression levels of MK‐related genes, such as *RUNX1*, *MPL*, *PF4* and *THBS1*, in the DC group (Figure 6H). Moreover, FCCP treatment inhibited the generation of CD41a^+^CD61^+^ cells from hESCs on Day 6 (Figure 6I). Consistently, the number of total CFUs and CFU‐MKs in the DC and SC groups was highly reduced after FCCP treatment (Figure 6J). Collectively, these data indicate that enhanced mitochondrial function caused by DC contributes to the skewing of hESCs toward megakaryopoiesis in vitro (Figure 7).

## 4 DISCUSSION

Recently, the transfusion of stem cell‐derived MK or platelet products has been developed as a potential therapeutic procedure to treat patients at risk of bleeding.^4^, ^30^ Pluripotent stem cells, such as hESCs or hiPSCs, are the most promising cell resources for in vitro MK or platelet product preparation owing to their extremely strong expansion and three‐germ‐layer differentiation capacity.^4^, ^5^ The scalable generation of MKs or PLTs from human pluripotent stem cells without gene modification is a big challenge and needs improvement.^4^, ^5^ Eto's lab has established expandable MKs from iPSCs and made great progress by using turbulence‐controllable bioreactors to promote platelet biogenesis.^31^ However, the induction technique for in vitro large‐scale generation of MKs from pluripotent stem cells still needs to be developed and improved. Since the expansion and differentiation processes of MKs derived from hESCs can be manipulated in a suspension culture manner,^18^ we aimed to further improve the yield of MKs derived from hESCs by introducing a bioreactor‐like manipulation protocol.

Increasing evidence has demonstrated various benefits of the dynamic baffled‐flow bioreactor system for the expansion of different types of cells owing to their scalability, improved oxygen transfer, and higher culture viability.^22^, ^32^, ^33^ We also found that the baffled‐flow culture system enabled a more efficient generation of CD41a^+^ and CD41a^+^CD42b^+^ MKs than the unbaffled‐flow culture system. By testing the relationship between the MK output and the rotary speeds of the orbital rotator, we further determined that the DC condition at 95 rpm was optimal for improving the yield of MKs derived from hESCs. The proper rotary speed created by the orbital rotator is critical for the cell spheroid size and yield.^34^ We found that lower or higher rotary speeds affected the formation of uniform cell spheroids and could not maintain hESC‐derived spheroids in the suspension growth state. Several studies have indicated that an inhomogeneous or larger size of EBs or colonies limits the differentiation efficiency of ESCs into target cells.^35^, ^36^, ^37^ Our dynamic culture system with a rotary speed of 95 rpm produced cell aggregates with a diameter of approximately 200.6 ± 72.6 μm during the induction process, which might help in efficient expansion and definite differentiation of hESCs toward MKs under haematopoietic and MK induction conditions. We found that this specific baffled‐flow culture system drastically enhanced the generation of MKs from both H1 and H9 hESCs (Figure S4A). Our data also indicated that modulation of hydrodynamic parameters of the culture medium may be used as a novel route to enhance MK production within suspension DC systems.^38^ This 3D dynamic suspension induction protocol could lead to a yield of 56.6 ± 30.7 MKs per H9 hESC by Day 12, which is significantly higher than the SC system. Prolonging the culture period may further increase the yield of MKs from hESCs.^39^, ^40^ In our experiments, we obtained 7.65 ± 2.83 × 10^7^ CD41a^+^ MKs from 1 × 10^6^ hESCs in a 125‐mL flask that was supplemented with 30 mL of medium. Theoretically, one 1‐L flask that was supplemented with up to 425 mL medium with baffled‐flow culture produced approximately 1 × 10^9^ CD41a^+^ MKs from hESCs after a 12‐day induction period. It is feasible to use this type of culture system to generate a large quantity of MKs that can be easily applied to bioreactors.^41^

The cells generated after the orderly three‐stage induction steps in our culture system were shown to have the characteristics of MK cells according to several evaluation criteria, including MK‐like cell morphology and ultrastructural features and the expression of key MK genes and surface markers. hESC‐derived MKs in the specific dynamic induction system displayed relatively mature characteristics compared with differentiated cells in the static induction method. MKs derived from the DC group showed stronger MK‐characteristic gene expression and an increased percentage of β1‐Tubulin^+^CD42^+^ cells. In addition, the development and maturation of MKs were accompanied by other unique changes, including gradually increasing cell size and granularity, which can be assessed based on FSC and SSC/granularity, respectively, using flow cytometry.^25^, ^42^ We found that the percentage of HG CD42b^+^ MKs in the DC group was significantly higher than that in the SC group (Figure S4B). Several studies have indicated that platelet‐producing human stem cell‐derived MKs can be identified and enriched using FV uptake.^25^ In our study, we found that HG MKs generated under specific DC conditions presented higher levels of FV uptake than those generated under SC conditions, indicating that the DC condition facilitated the generation of platelet‐producing MKs. Moreover, these differentiated MKs could further develop and differentiate into proplatelets and PLTs after another two‐stage induction, further supporting their function in platelet release.

We then explored the possible mechanism by which specific DC conditions enhance megakaryopoiesis in hESCs. We found that MK fate was significantly facilitated during the early haematopoiesis process initiated from hESCs under this specific DC condition, as evidenced by increased percentages and number of CFU‐MKs in the total CFUs, enhanced expression of MK‐related genes, and cell surface markers. Furthermore, the RNA‐seq data and experimental results indicated that OXPHOS and mitochondrial function were significantly enhanced under DC conditions. Both CD34^+^CD43^+^ haematopoietic cells and CD34^−^CD43^+^ blood cells presented higher mean fluorescence intensity of MitoTracker than CD34^+^CD43^−^CD73^−^ HE (Figure S5A), suggesting that the mitochondrial mass in the generated haematopoietic cells was increased under DC conditions. Interestingly, induced cells from the DC group had higher mitochondrial reactive oxygen species levels than those from the SC group (Figure S5B), which might be due to the enhanced OXPHOS pathway under specific conditions.^43^ Additionally, we verified that the improved mitochondrial activity by the specific DC condition was markedly impaired by the addition of FCCP, confirming that megakaryopoiesis requires better mitochondrial activity to promote the expression of key MK genes and cell surface markers.^44^ Relative to other strategies for improving mitochondrial function by introducing cocultured cells or nanoparticles,^45^, ^46^ the baffled‐flow DC manner greatly simplified the cell product manufacturing process via physically modulating mitochondrial activity. Oburoglu et al. reported that OXPHOS‐mediated differentiation of HE toward definitive haematopoiesis is dependent on cholesterol metabolism.^47^ It would be meaningful to further investigate the metabolic pathways that regulate megakaryopoiesis.

In summary, we developed a baffled‐flow culture system and found that this system significantly promoted the efficient and mass generation of MKs derived from hESCs within 12 days. Our data indicate that it is worthwhile to further develop baffled‐flow shaken bioreactors for the scale‐up of MK manufacturing from hESCs in suspension. Indeed, this DC condition drives MK fate commitment during the early haematopoietic process in hESCs. Mechanistically, we found that enhancing OXPHOS and mitochondrial function in this specific culture manner mainly contributed to the skewing of hESCs toward megakaryopoiesis. This study could aid in the automatic and scalable production of MKs from stem cells using baffled‐flow bioreactors and could also assist in the manufacture of hESC‐derived MK and platelet products.

## AUTHOR CONTRIBUTIONS

The contributions of each author made to the study are specified as follows: Yanhua Li and Xuetao Pei designed the study; Xumin Wu, Bowen Zhang, Keyi Chen, Jiahui Zhao, Yunxing Li, Jisheng Li, Chuanli Liu, Lijuan He, Tao Fan and Chao Wang performed the experiments and collected the data; Xumin Wu, Bowen Zhang, Yanhua Li and Yan Li analysed the data; Yanhua Li, Xumin Wu and Bowen Zhang prepared the manuscript. All authors have read and approved the manuscript.

## CONFLICT OF INTEREST STATEMENT

The authors declare that they have no competing interests.

## Supporting information


**Table S1.** The composition of BEL medium
**Table S2.** Key resources table
**Table S3.** Primer

**Figure S1.** Comparison of DC for in vitro megakaryopoiesis of hESCs. (A and B) Bar graph showing the percentage and number of CD41a^+^ and CD41a^+^CD42b^+^ cells after induction in unbaffled flasks (UBF) or baffled flasks (BF) for 12 days. *n* = 3. (C) Fold of CD41a^+^ cells to hESCs during induction in BF at different rotation speeds (0, 60, 95, 130 rpm). *n* = 3.

**Figure S2.** Expression of mesodermal progenitor marker APLNR was observed at stage I. *n* = 3.

**Figure S3.** Baffled‐flow culture promotes the generation of MK‐fated haematopoiesis. (A) Volcano plot displaying DEGs of CD34^+^ cells in SC and DC groups on Day 6. (B) KEGG analysis indicates the top‐20 terms enriched by the upregulated genes in DC groups compared to SC groups on Day 6. The size of each dot is based on the number of genes enriched in the pathway, and the colour of the dots represents the significance of pathway enrichment. (C) GSEA comparing hESC‐derived CD34^+^ cells on Day 6 from SC and DC groups for platelet degranulation and coagulation gene set. (D) Representative flow cytometry results of surface marker CD34, CD41a and CD42b after haematopoietic differentiation assays for 5 days.

**Figure S4.** Baffled‐flow culture system produced mass hESC‐derived MKs. (A) Fold of MKs to H1 or H9 during differentiation under SC and DC conditions on Day 12. *n* = 8 for H1‐MKs, *n* = 6 for H9‐MKs. (B) Bar graph showing the percentage of LG CD42b^+^ and HG CD42b^+^ MKs in SC and DC groups. *n* = 3.

**Figure S5.** Generation of haematopoietic cells was accompanied by an increase in mitochondrial abundance. (A) Changes of Mito‐Tracker during endothelium‐to‐haematopoietic transition. (B) Relative fluorescence of MitoSOX/Mito‐Tracker of hESC‐derived CD34^+^ cells in SC and DC groups. *n* = 3.

## Data Availability

The authors confirm that the data supporting the findings of this study are available within the published article and its supplementary files.
