# Supplementary material for: Baffled‐flow culture system enables the mass production of megakaryocytes from human embryonic stem cells by enhancing mitochondrial function
Source: Cell Prolif. 2023 Apr 23;56(12):e13484. doi: 10.1111/cpr.13484 (PMC10693187; doi:10.1111/cpr.13484)
Supplement: Supplementary file 1 — Table S1. The composition of BEL medium Table S2. Key resources table Table S3. Primer [file CPR-56-e13484-s005.docx]

**Supplemental Methods**

**Supplemental Tables**

**Table S1** **The composition of BEL medium**

| **Media Component** | **Component Content** |
| --- | --- |
| IMDM/F-12 | 1:1 |
| Deionized BSA | 2.5 mg/mL |
| Synthechol Solution | 0.2 % |
| Linoleic Acid | 100 ng/mL |
| Linolenic Acid | 100 ng/mL |
| Ascorbic Acid 2-phosphate | 50 μg/mL |
| α-MTG | 450 μM |
| GlutaMAX | 2 mM |
| Protein-Free Hybridoma Mix | 5 % |
| Insulin-Transferrin-Selenium | 1 % |

**Table S2. Key Resources Table**

| **Antibodies** | **Source** | **Identifier** |
| --- | --- | --- |
| Anti-hCD41-BV421 | Biolegend | Cat#303730 |
| Anti-hCD41a-PE | eBioscience | Cat#12-0419-42 |
| Anti-hCD41a-APC | eBioscience | Cat#17-0419-42 |
| Anti-hCD41a-FITC | eBioscience | Cat#11-0419-42 |
| Anti-hCD61-FITC | eBioscience | Cat#11-0619-42 |
| Anti-hCD42b-PE | eBioscience | Cat#12-0429-42 |
| Anti-hCD42b-APC | eBioscience | Cat#17-0429-42 |
| Anti-hCD73-FITC | eBioscience | Cat#11-0739-42 |
| Anti-hCD34-PECy7 | BD | Cat#560710 |
| Anti-hCD45-BV786 | BD | Cat#563716 |
| Anti-hCD43-BV421 | BD | Cat#562916 |
| Anti-hCD144-Percp | BD | Cat#561566 |
| Hoechst 33342 Solution | BD | Cat#561908 |
| Fixable Viability Stain 510 (FVS 510) | BD | Cat#564406 |
| Mouse anti-CD42b | BD | Cat#555471 |
| Rabbit anti-VWF | Millipore | Cat#AB7356 |
| Mouse anti-β1-Tubulin | R&D Systems | Cat#MAB8527 |
| Rabbit anti-CD41 | Abclonal | Cat#A11490 |
| Rabbit anti-GP1BA | Abclonal | Cat#A16048 |
| Atto 488 Conjugation Kit | abcam | Cat#ab269896 |
| Atto 633 Conjugation Kit | abcam | Cat#ab269898 |
| Donkey Anti-Mouse IgG H&L (Alexa Fluor® 488) | abcam | Cat#ab150105 |
| Donkey Anti-Rabbit IgG H&L (Alexa Fluor® 568) | abcam | Cat#ab175470 |
|  |  |  |
| **Chemicals and Recombinant Proteins** | **Source** | **Identifier** |
| Recombinant Human SCF | Peprotech | Cat#300-07 |
| Recombinant Human Flt3-Ligand | Peprotech | Cat#300-19 |
| Recombinant Human IL-3 | Peprotech | Cat#200-03 |
| Recombinant Human TPO | Peprotech | Cat#300-18 |
| Recombinant Human FGF-basic | Peprotech | Cat#100-18c |
| Human IL-11 | Peprotech | Cat#200-11 |
| Human RANTES (CCL5) | Peprotech | Cat#300-06 |
| Recombinant Human BMP-4 Protein | R&D Systems | Cat#314-BP |
| Recombinant Human Activin A Protein | R&D Systems | Cat#338-AC |
| Recombinant Human VEGF Protein | R&D Systems | Cat#293-VE |
| Recombinant Human IGF-1 Protein | R&D Systems | Cat#291-G1 |
| Matrigel^®^ Matrix | Corning | Cat#354277 |
| SB431542 | Selleck | Cat#S1067 |
| CHIR99021 | Selleck | Cat#S2924 |
| Y27632 | Selleck | Cat#S1049 |
| Q-VD-Oph | Selleck | Cat#S7311 |
| StemRegenin 1 (SR1) | MedChemExpress | Cat#[HY-15001](https://www.medchemexpress.cn/StemRegenin-1.html) |
| KP-457 | MedChemExpress | Cat#HY-110397 |
| TA-316 | MedChemExpress | Cat#HY-112486 |
| Prostaglandin E_1_ ( PGE_1_) | MedChemExpress | Cat#HY-B0131 |
| Harmine | MedChemExpress | Cat#[HY-N0737A](https://www.medchemexpress.cn/Harmine.html) |
| dimethylfasudil (diMF) | Merck | Cat#555550 |
| Human Coagulation factor V (FV) | CUSABIO | Cat#CSB-EP007929HU |
|  |  |  |
| **Culture Medium** | **Source** | **Identifier** |
| mTeSR1 | Stemcell Technologies | Cat#85850 |
| Advanced DMEM/F-12 | Gibco | Cat#12634010 |
| F12＋GlutaMAX | Gibco | Cat#31765035 |
| IMDM＋GlutaMAX | Gibco | Cat#31980030 |
|  |  |  |
| **Reagent or Resource** | **Source** | **Identifier** |
| Ascorbic Acid 2-phosphate (AA2P) | Sigma-Aldrich | Cat#A8960 |
| Deionized BSA | Sigma-Aldrich | Cat#A3311 |
| Synthechol Solution | Sigma-Aldrich | Cat#S-5442 |
| Linolenic Acid | Sigma-Aldrich | Cat#L0288 |
| Linoleic Acid | Sigma-Aldrich | Cat#L1012 |
| α-MTG | Sigma-Aldrich | Cat#M6145 |
| GlutaMAX | Gibco | Cat#35050061 |
| Protein-Free Hybridoma Mix (PFHM-Ⅱ) | Gibco | Cat#12040027 |
| Insulin-Transferrin-Selenium (ITS) | Gibco | Cat#41400045 |
| Chemically Defined Lipid Concentrate | Gibco | Cat#11905031 |
| Heparin Solution | STEMCELL Technologies | Cat#07980 |
| MethoCult^TM^ SF4636 | STEMCELL Technologies | Cat#04636 |
| MethoCult^TM^ H4535 Enriched Without EPO | STEMCELL Technologies | Cat#04535 |
| MegaCult™-C Complete Kit with Cytokines | STEMCELL Technologies | Cat#04971 |
| Mitochondrial Membrane Potential Assay with TMRE | Beyotime | Cat#C2001S |
| ATP Assay Kit | Beyotime | Cat#S0026 |
| Mito-Tracker Green | Beyotime | Cat#C1048 |
| MitoSOX™ Red | Invitrogen™ | Cat#M36008 |
| CD34 MicroBead Kit | Miltenyi Biotec | Cat#130-046-702 |

**Table S3. Primer**

| **Gene Name** | **Forward** | **Reverse** |
| --- | --- | --- |
| *TIE1* | AAGCAGACAGACGTGATCTGG | GCACGATGAGCCGAAAGAAG |
| *ERG* | CGTGCCAGCAGATCCTACG | GGTGAGCCTCTGGAAGTCG |
| *ETV2* | GAAGGAGCCAAATTAGGCTTCT | GAGCTTGTACCTTTCCAGCAT |
| *SCL* | AGCCGGATGCCTTCCCTAT | GGGACCATCAGTAATCTCCATCT |
| *MYB* | GAAAGCGTCACTTGGGGAAAA | TGTTCGATTCGGGAGATAATTGG |
| *GATA2* | GCAACCCCTACTATGCCAACC | CAGTGGCGTCTTGGAGAAG |
| *RUNX1* | CTGCCCATCGCTTTCAAGGT | GCCGAGTAGTTTTCATCATTGCC |
| *THBS1* | AGACTCCGCATCGCAAAGG | TCACCACGTTGTTGTCAAGGG |
| *β1-TUBULIN* | AACACGGGATCGACTTGGC | CTCGGGGCACATATTTCCTAC |
| *PF4* | AGCCTGGAGGTGATCAAGG | CCATTCTTCAGCGTGGCTA |
| *MPL* | CTGAAGTGTTTCTCCCGAACAT | GCGGGTAGGCATACAGCAG |
| *GFI1B* | GCAGGAAGATGAACCGCTCT | CCAGGCACTGGTTTGGGAA |
| *MEIS1* | GGGCATGGATGGAGTAGGC | GGGTACTGATGCGAGTGCAG |
| *FLI1* | CAGCCCCACAAGATCAACCC | CACCGGAGACTCCCTGGAT |
| *ZFPM1* | CGTGCTTCGAGTGCGAGAT | GGCCTGAACAGTAGAGGCG |
| *NFE2* | GCAGGAACAGGGTGATACAGC | GCAGCTCGGTGATGGACAT |
| *GAPDH* | GAGTCAACGGATTTGGTCGT | TTGATTTTGGAGGGATCTCG |

**Supplemental Figure Legend**

**Figure S1.** Comparison of DC for *in vitro* megakaryopoiesis of hESCs. A and B, Bar graph showing the percentage and number of CD41a^+^ and CD41a^+^CD42b^+^ cells after induction in unbaffled flasks (UBF) or baffled flasks (BF) for 12 days. n = 3. C, Fold of CD41a^+^ cells to hESCs during induction in BF at different rotation speeds (0, 60, 95, 130 rpm). n = 3.

**Figure S2.** Expression of mesodermal progenitor marker APLNR was observed at stage Ⅰ. n = 3.

**Figure S3.** Baffled-flow culture promotes the generation of MK-fated hematopoiesis. A, Volcano plot displaying DEGs of CD34^+^ cells in SC and DC groups on day 6. B, KEGG analysis indicating the top-twenty terms enriched by the upregulated genes in DC groups compared to SC groups on day 6. The size of each dot is based on the number of genes enriched in the pathway, and the color of the dots represents the significance of pathway enrichment. C, GSEA comparing hESC-derived CD34^+^ cells on day 6 from SC and DC groups for platelet degranulation and coagulation gene set. D, Representative flow cytometry results of surface marker CD34, CD41a and CD42b after hematopoietic differentiation assays for 5 days.

**Figure S4.** Baffled-flow culture system produced mass hESC-derived MKs. A, Fold of MKs to H1 or H9 during differentiation under SC and DC conditions on day 12. n = 8 for H1-MKs, n = 6 for H9-MKs. B, Bar graph showing the percentage of LG CD42b^+^ and HG CD42b^+^ MKs in SC and DC groups. n = 3.

**Figure S5.** Generation of hematopoietic cells was accompanied with an increase in mitochondrial abundance. A, Changes of Mito-Tracker during endothelial-to-hematopoietic transition. B, Relative fluorescence of MitoSOX/Mito-Tracker of hESC-derived CD34^+^ cells in SC and DC groups. n = 3.
