# Supplementary figures and images for: Baffled‐flow culture system enables the mass production of megakaryocytes from human embryonic stem cells by enhancing mitochondrial function
Source: Cell Prolif. 2023 Apr 23;56(12):e13484. doi: 10.1111/cpr.13484 (PMC10693187; doi:10.1111/cpr.13484)

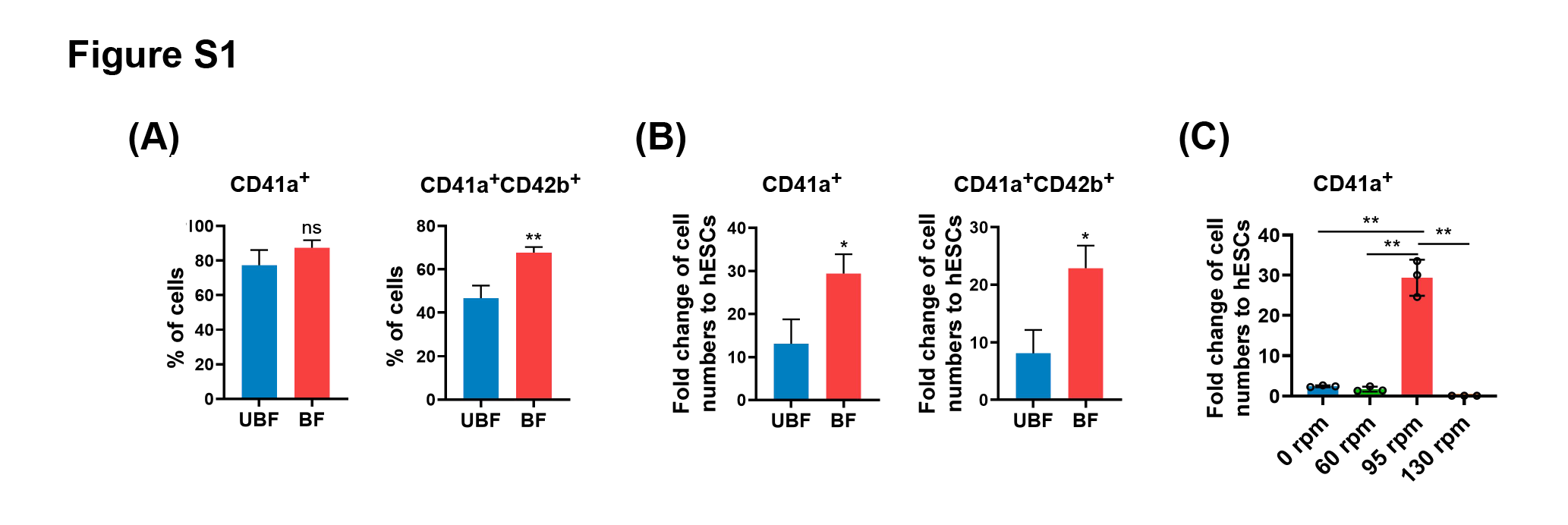

Supplement: Supplementary file 2 — Figure S1. Comparison of DC for in vitro megakaryopoiesis of hESCs. (A and B) Bar graph showing the percentage and number of CD41a+ and CD41a+CD42b+ cells after induction in unbaffled flasks (UBF) or baffled flasks (BF) for 12 days. n = 3. (C) Fold of CD41a+ cells to hESCs during induction in BF at different rotation speeds (0, 60, 95, 130 rpm). n = 3. [file CPR-56-e13484-s001.tif]

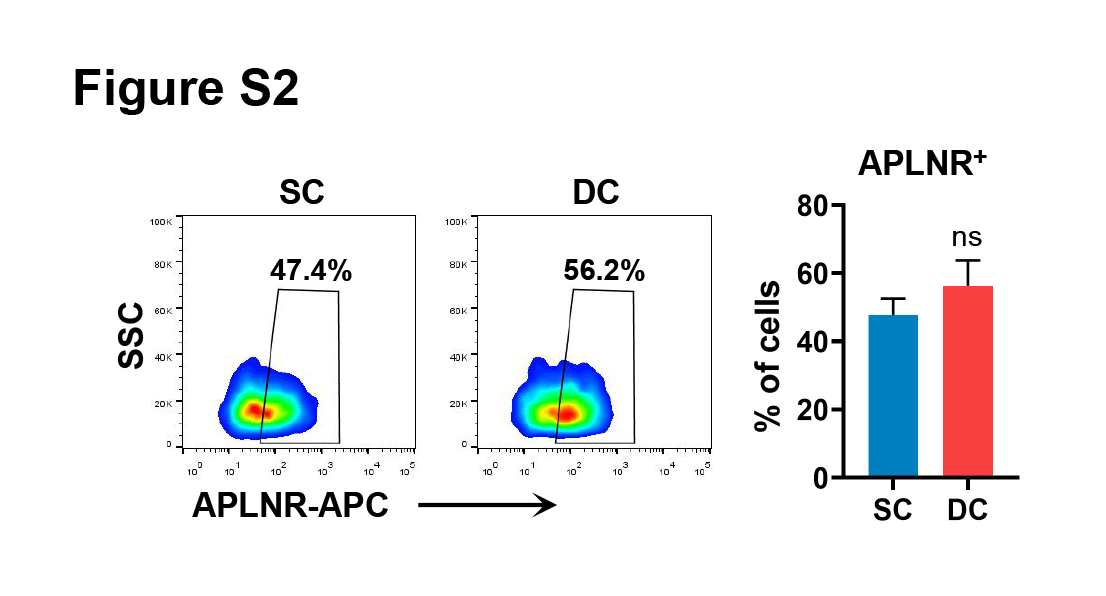

Supplement: Supplementary file 3 — Figure S2. Expression of mesodermal progenitor marker APLNR was observed at stage I. n = 3. [file CPR-56-e13484-s003.tif]

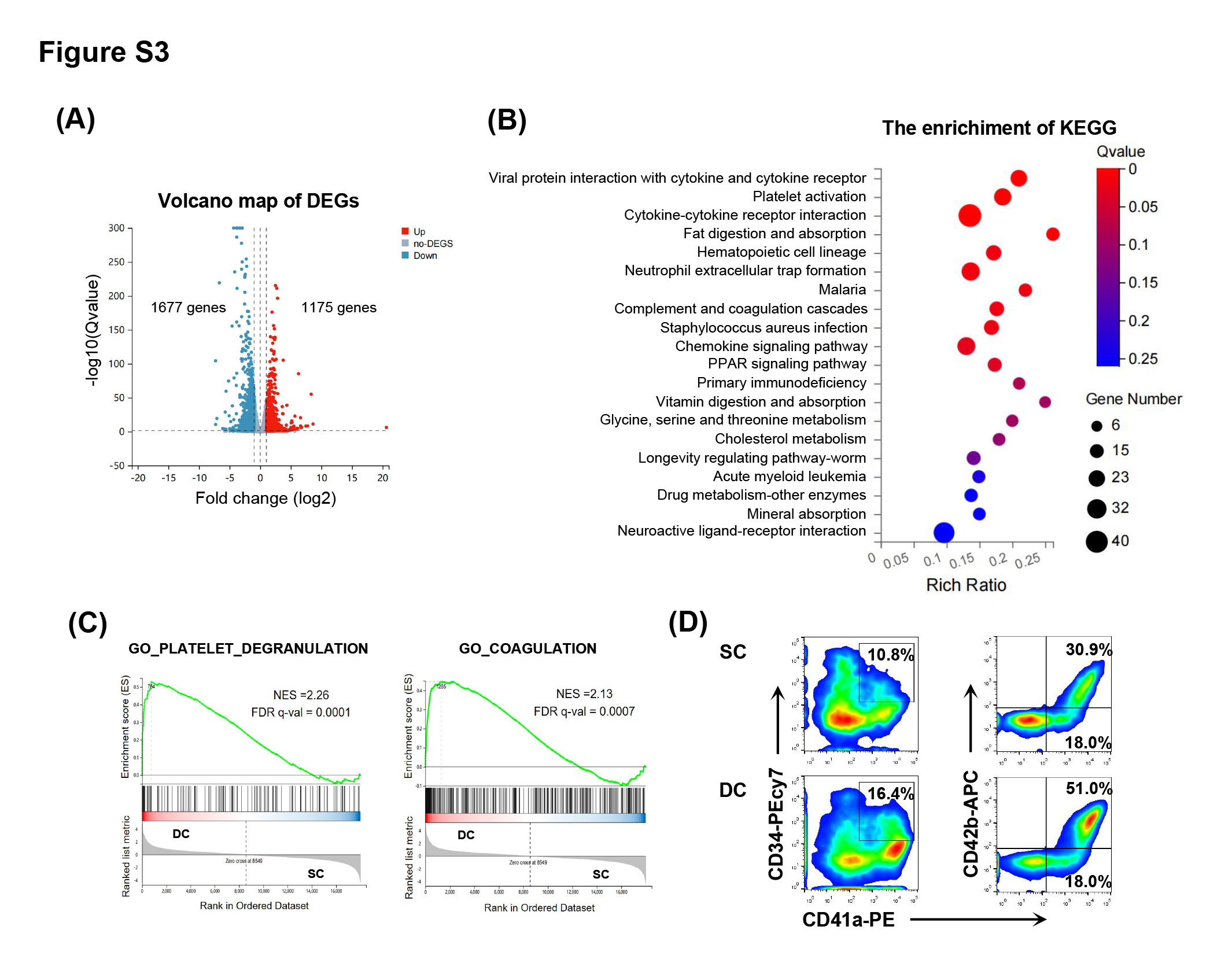

Supplement: Supplementary file 4 — Figure S3. Baffled‐flow culture promotes the generation of MK‐fated haematopoiesis. (A) Volcano plot displaying DEGs of CD34+ cells in SC and DC groups on Day 6. (B) KEGG analysis indicates the top‐20 terms enriched by the upregulated genes in DC groups compared to SC groups on Day 6. The size of each dot is based on the number of genes enriched in the pathway, and the colour of the dots represents the significance of pathway enrichment. (C) GSEA comparing hESC‐derived CD34+ cells on Day 6 from SC and DC groups for platelet degranulation and coagulation gene set. (D) Representative flow cytometry results of surface marker CD34, CD41a and CD42b after haematopoietic differentiation assays for 5 days. [file CPR-56-e13484-s004.tif]

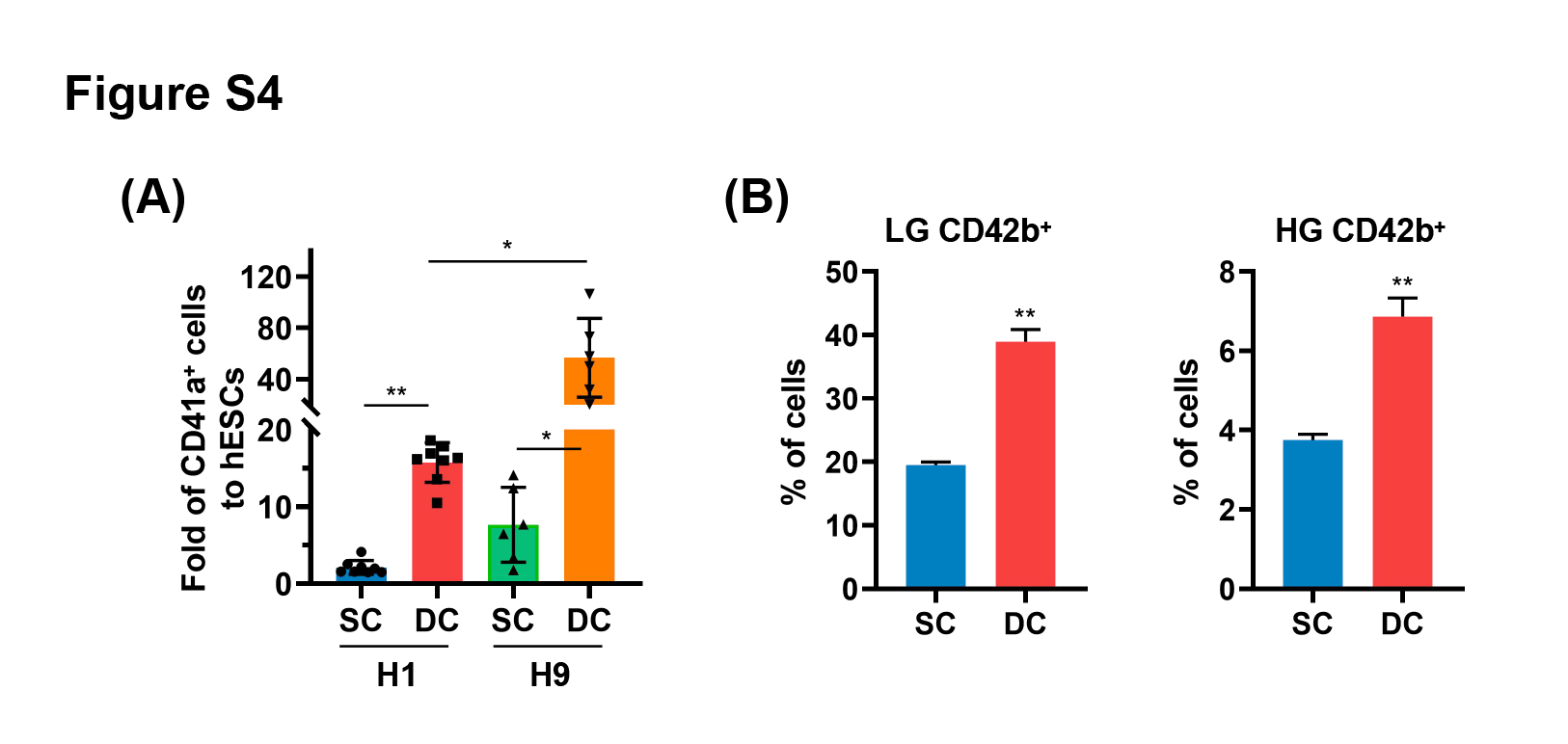

Supplement: Supplementary file 5 — Figure S4. Baffled‐flow culture system produced mass hESC‐derived MKs. (A) Fold of MKs to H1 or H9 during differentiation under SC and DC conditions on Day 12. n = 8 for H1‐MKs, n = 6 for H9‐MKs. (B) Bar graph showing the percentage of LG CD42b+ and HG CD42b+ MKs in SC and DC groups. n = 3. [file CPR-56-e13484-s002.tif]

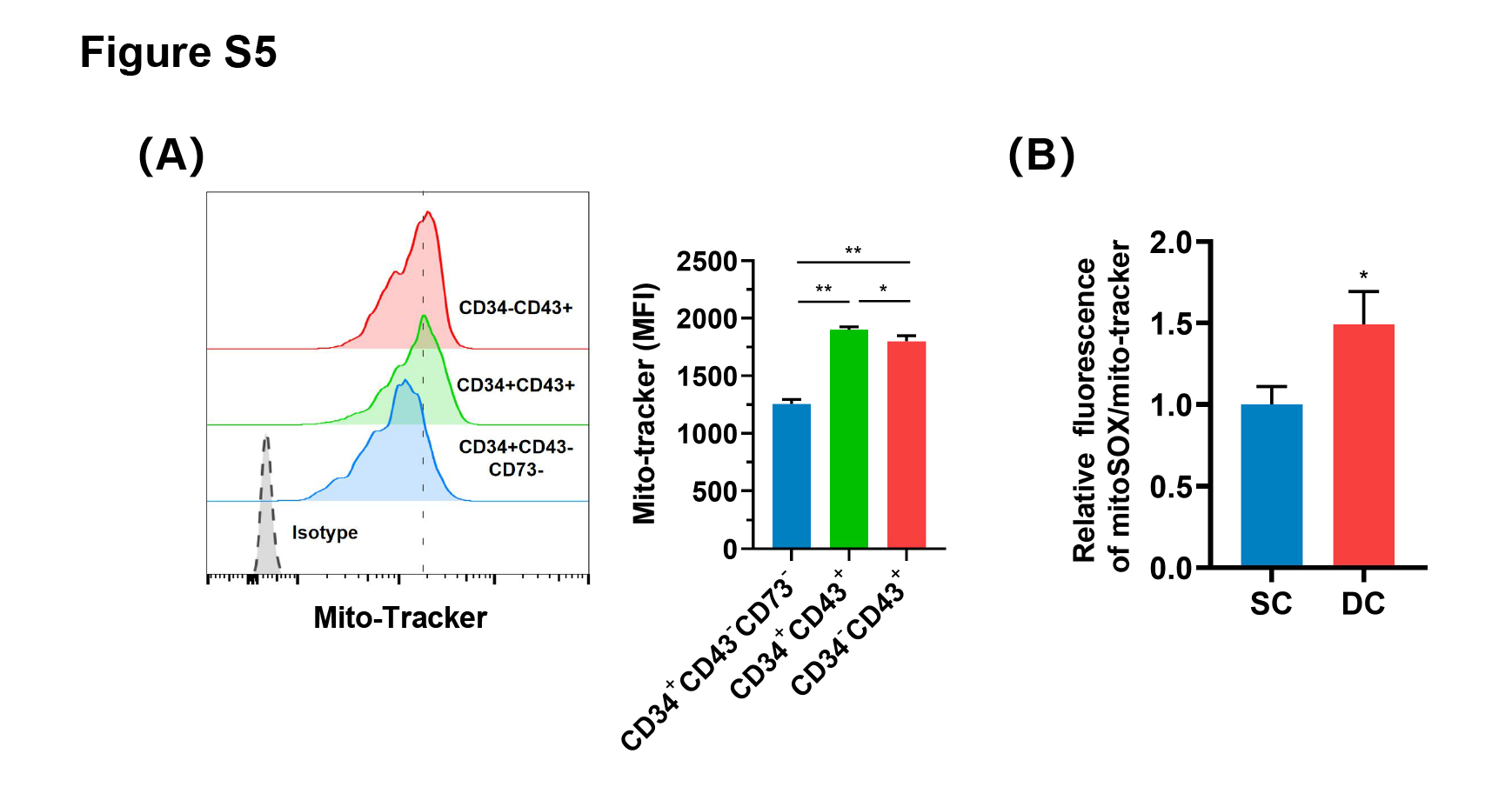

Supplement: Supplementary file 6 — Figure S5. Generation of haematopoietic cells was accompanied by an increase in mitochondrial abundance. (A) Changes of Mito‐Tracker during endothelium‐to‐haematopoietic transition. (B) Relative fluorescence of MitoSOX/Mito‐Tracker of hESC‐derived CD34+ cells in SC and DC groups. n = 3. [file CPR-56-e13484-s006.tif]
